# Implant Deviation Assessment in Static and Dynamic Guidance: An In Vitro Study

**DOI:** 10.7759/cureus.105355

**Published:** 2026-03-17

**Authors:** Charbel Kachouh, Stephanie Mrad, Ronald Younes, Hani Tohme, Nabil Ghosn, Adam Saleh, Abdallah Menhall

**Affiliations:** 1 Department of Oral Surgery, Faculty of Dental Medicine, Saint Joseph University of Beirut, Beirut, LBN; 2 Department of Digital Dentistry, AI, and Evolving Technologies, Faculty of Dental Medicine, Saint Joseph University of Beirut, Beirut, LBN; 3 Laboratory of Craniofacial Research, Faculty of Dental Medicine, Saint Joseph University of Beirut, Beirut, LBN; 4 Department of Periodontology, Faculty of Dental Medicine, Saint Joseph University of Beirut, Beirut, LBN

**Keywords:** accuracy, computer-assisted surgery, dental implant, surgical navigation systems, three-dimensional printing

## Abstract

Purpose

This in vitro study aims to assess implant placement positioning accuracy using static or dynamic guidance by measuring 3D, 2D, and volumetric deviations of 20 implants compared to the reference implant position obtained following preoperative implant planning.

Methods

A case of single-tooth edentulism was used to simulate the situation of single implant placement. A cone beam computed tomography (CBCT) scan (Orthophos SL 3D, Dentsply Sirona, Mölndal, Sweden) of the model was taken, and implant planning was done on the Blue Sky Plan (BSP) software (Blue Sky Bio, New York City, NY), generating an implant reference position. The 20 printed models were randomly divided into two equal groups, with both receiving implant placement using either static guidance using polymethylmethacrylate (PMMA)-printed templates (group 1) or dynamic guidance using the X-Guide^® ^(X-Nav Technologies, LLC, Lansdale, PA, USA) navigation system (group 2). A high-precision non-reflective abutment-level scan body was mounted on the implant directly after placement, and a surface scan was taken. The reference implant and placed implant standard tessellation language (STL) files were aligned using the Medit Link^® ^software (Medit, Seoul, South Korea). Three-dimensional and 2D deviations were assessed using the Geomagic^®^ Control X software (3D Systems, Rock Hill, SC), and volumetric deviation was assessed using the Blender™ software (Blender Foundation, Amsterdam, Netherlands). Statistical analysis was performed using RStudio version 2024.12.1 (Posit Software, Boston, MA).

Results

Twenty scans were obtained in total (n = 10 per group). The significance level was set at p ≤ 0.05. Statistical analysis revealed statistical significance in terms of 3D coronal deviation (p < 0.001), coronal linear deviation (CLD) (p = 0.036), coronal vertical deviation (CVD) (p < 0.001), and apical vertical deviation (AVD) (p = 0.001). However, 3D apical deviation (p = 0.131), apical linear deviation (ALD) (p = 0.945), angular deviation (p = 0.097), and accuracy percentage (p = 0.954) did not reach statistical significance.

Conclusion

Dynamic guidance showed higher coronal and vertical deviations compared to static guidance, while overall accuracy and global implant deviation remained comparable between the techniques. This in vitro study consisted of resin models lacking bone density or gingival tissue simulation, which limits the extrapolation of the findings to clinical practice. With that being said, further validation of these results through clinical studies involving a larger sample is required.

## Introduction

Currently, implant-based therapies, with success rates of up to 95% [1], can be considered a predictable and safe masticatory rehabilitation treatment for partially or fully edentulous patients [2]. Despite recent advancements in implantology, complications can still occur [3]. According to the International Team for Implantology (ITI), these complications are multifactorial and depend on four main factors: the patient, biomaterial, clinician, and treatment approach [4]. The primary responsibility for evaluating site and patient factors lies with the clinician [5], as the lack of preoperative planning [6], such as improper implant-implant or implant-tooth distance during implant placement [7], can increase complication incidence. These complications can be classified into three correlated categories: biological, surgical, and prosthetic [8]. Over the years, conventional, or freehand implant placement (FHIP), has been the most adopted method for implant placement [9]. However, many complications, such as direct root invasions and root surface proximity, have been reported following this technique [10]. To decrease the likelihood of such adverse events, guided implant placement (GIP) has been suggested as an alternative to FHIP [11].

There are two documented types of GIP: static-guided implant placement (s-GIP) and dynamic navigation or dynamic-guided implant placement (d-GIP) [12]. s-GIP templates are placed on the surgical site, facilitating implant placement according to the previously planned position [13]. As per d-GIP, it benefits from interactive guidance, in-synch drilling instrument, and implant state monitoring at the surgical site [14]. During the procedure, tracking cameras are implemented to continuously monitor markers attached to the patient's jaw and surgical drilling handpiece via a patient-tailored thermoplastic device [15]. Procedure progress is displayed in real time on a built-in monitor, allowing any drill or implant deviation to be detected and corrected according to preoperative virtual planning. However, d-GIP efficacy may be affected by several factors such as system calibration, tracker stability, and operator experience, all of which are important when interpreting the results of this guidance technique [15].

This study aims to assess and compare implant deviations in s-GIP and d-GIP by studying the positioning accuracy of an implant placed in a bounded edentulous area in standardized models under controlled conditions. The primary endpoint is to assess which guided technique leads to less overall implant deviation when compared to the planned implant position. The secondary objective is to evaluate the accuracy of each guided technique. The null hypothesis (H_0_) is that s-GIP and d-GIP do not exhibit statistically significant overall 2D, 3D, and volumetric implant deviations when compared to the initial planned implant position. Compared with conventional 2D and 3D measurement, volumetric analysis was used as a novel endpoint to allow a more global quantification of spatial discrepancies. The alternative hypothesis (H_1_) suggests that s-GIP and d-GIP exhibit statistically significant overall implant deviations when compared to the initial planned implant position.

## Materials and methods

Pre-implant placement

**Figure 1:**
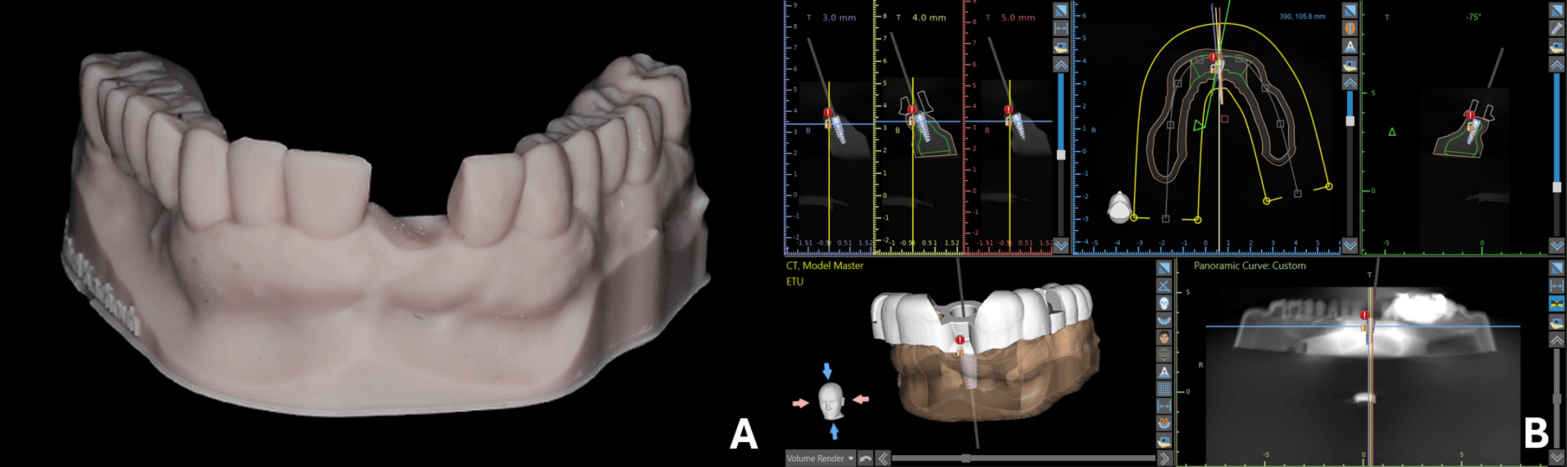
(A) Printed model; and (B) prosthetically driven implant planning on BSP BSP: Blue Sky Plan; ETU: Edentulous single Tooth Unit.

The protocol of thisin vitroexperimental study was accepted by the Saint Joseph University of Beirut Research Ethics Committee, Beirut, Lebanon (approval number: Tfemd-2025-38, dated: December 10, 2024). A virtual model simulating a single-tooth replacement implant scenario was digitally designed. The model standard tessellation language (STL) file was transferred to the digital light projector (DLP) 3D printing machine Elegoo^®^ Saturn 4 Ultra (Elegoo Technology Co., Ltd., Shenzhen, China) using "Dentratec Model" (CUBE 3D Printing Experts, Beirut, Lebanon) resin (Figure 1A). Twenty replicas were printed and randomly divided into two equal groups comprising 10 models each. The study also comprised 20 implants (10 per group), consistent with similarin vitro investigations. The model underwent a cone beam computed tomography (CBCT) scan (Orthophos SL 3D, Dentsply Sirona, Mölndal, Sweden), generating a digital imaging and communications in medicine (DICOM) file for the model. Model STL and DICOM files were then exported to Blue Sky Plan (BSP) version 4.13.35 (Blue Sky Bio, New York City, NY) and aligned, allowing for prosthetically driven implant planning (Figure 1B). A 4.3x13 mm Nobel Biocare implant (Nobel Biocare^®^ AB, Göteborg, Sweden) was planned in a prosthetically driven position. Subsequently, a tooth-supported surgical guide was also designed on BSP.

Implant placement

s-GIP (Group 1)

**Figure 2:**
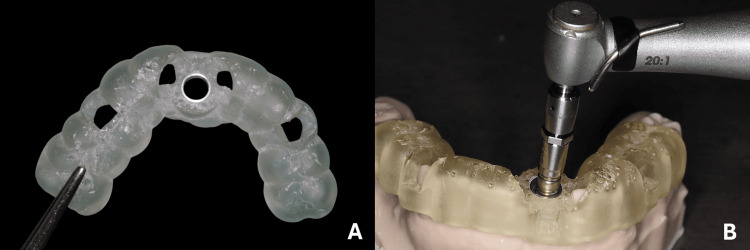
(A) Printed guide with the RP-guided sleeve; and (B) implant inserted through the guide upon placement

The surgical guide STL file was transferred to the liquid crystal display (LCD) 3D printer (Dentratec^®^, Beirut, Lebanon) for printing using polymethylmethacrylate (PMMA) resin (Voco GmbH, Germany). Ten guides were then printed, cleaned, trimmed, and cured. A guided sleeve RP (Nobel Biocare^® ^AB, Göteborg, Sweden) was press-fitted into the guides (Figure 2A). For the two groups, osteotomy was performed by a single highly experienced operator in both s-GIP and d-GIP. Printed surgical guides were placed on group 1 models using the corresponding guided implant placement protocol drills. Each osteotomy was realized using drill handles completely inserted through the guide, parallel to sleeve inner wall [16]. The drilling sequence followed manufacturer recommendations for s-GIP. Subsequent resin debris was removed after each drill passage using compressed air. A total of 10 implants were placed in all 10 group 1 models (Figure 2B).

d-GIP (Group 2)

**Figure 3:**
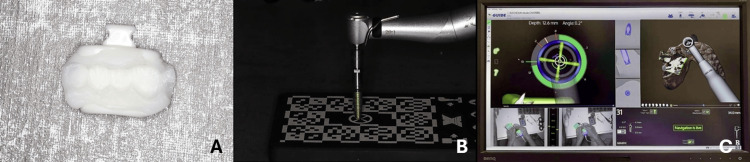
(A) X-Clip® with the imprint of the model teeth; (B) implant length measurement prior to placement; and (C) implant site preparation progression on X-Guide® (X-Nav Technologies, LLC, Lansdale, PA, USA)

Prior to the CBCT scan and as per manufacturer guidelines, a thermoplastic device with three radiopaque markers (X-Clip^®^, X-NAV Technologies, LLC, Lansdale, PA, USA) was placed on the model contralateral residual teeth. The device was warmed in hot water to 75°C for seven minutes, until soft and translucent. Once soft, it was removed from the water and positioned on the model teeth. Once cool to the touch, the device was removed vertically without any lateral movement and left until white, hard, and radiopaque. Device stability and position were assessed to confirm the absence of any undesired rocking movements (Figure 3A). The thermoplastic device was then positioned on the model.

In group 2, the calibration process was conducted in accordance with manufacturer recommendations, 60-80 cm below the tracking cameras. It started with the continuous movement of the contra-angle, along with its attached tracker, both being continuously moved under the cameras at a 30° upward tilt. Upon the completion of this step, the software automatically progressed to the "Disc Chuck Calibration" phase. The calibration disc and the motor were then attached to the contra-angle and rotated at a speed of 20 revolutions per minute (rpm), within the manufacturer's recommended range (10-25 rpm). Following this, the "X-Clip^® ^Device Calibration" phase was performed by continuously moving the device to ensure all markers were fully visible and detectable. Drill length measurement was then conducted by inserting the pilot drill (Ø = 2.0 mm) into the contra-angle, positioning it at the center of the "Go Plate" (Go Plate^®^, X-Nav Technologies, LLC, Lansdale, PA, USA), and measuring it accordingly. Subsequently, the "Calibration Check" step was performed by touching the three X-Clip^®^ fiducial markers with the drill to verify proper calibration. Calibration was considered successful if all values were green and below 0.20 mm. Each step was confirmed by the calibration completion indicator. Once the calibration process was finalized, the system advanced to the "Live Navigation" phase. Calibration and osteotomies were performed following the manufacturer's recommended sequence using the W&H model WI-75 E/KM contra-angle (W&H Dentalwerk, Salzburg, Austria), Nobel Biocare^®^ OsseoSet™ Type 200 motor (Nobel Biocare AG, Kloten, Switzerland), and NobelReplace CC PureSet drills (Nobel BioCare^®^ AB, Göteborg, Sweden). The drilling sequence involved measuring each drill length using the "Go Plate" before osteotomy preparation. Prior to implant placement, the implant was also placed on the "Go Plate" to measure its length (Figure 3B). After each drilling step, resin debris was removed using compressed air. A total of 10 implants were placed across all group 2 models, and implant site preparation progression was displayed on a monitor (Figure 3C).

Post-implant placement

**Figure 4:**
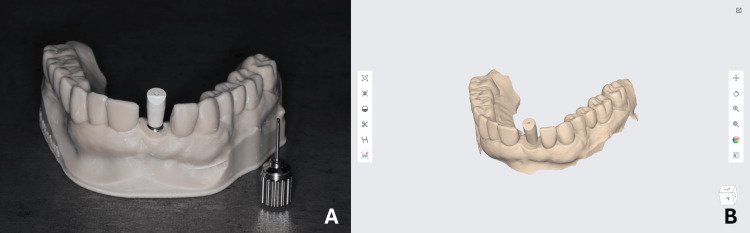
(A) Mounted scan body after implant placement; and (B) surface scan

After placing the implants in group 1 and 2 models, a high-precision non-reflective abutment-level scan body (IO 2B-B SA, Nobel BioCare^®^ AB, Göteborg, Sweden) was mounted on each implant and then hand-tightened not exceeding 5 N/cm, as per the manufacturer's instructions (Figure 4A). Following the manufacturer's scanning strategy, an optical surface scan using the Medit^®^ i700 (Medit, Seoul, South Korea) was taken for all 20 models directly after implant placement, generating an STL file for each. Impression was considered complete when no substantial voids were present, particularly in the scan body area (Figure 4B).

Variable assessment

**Figure 5:**
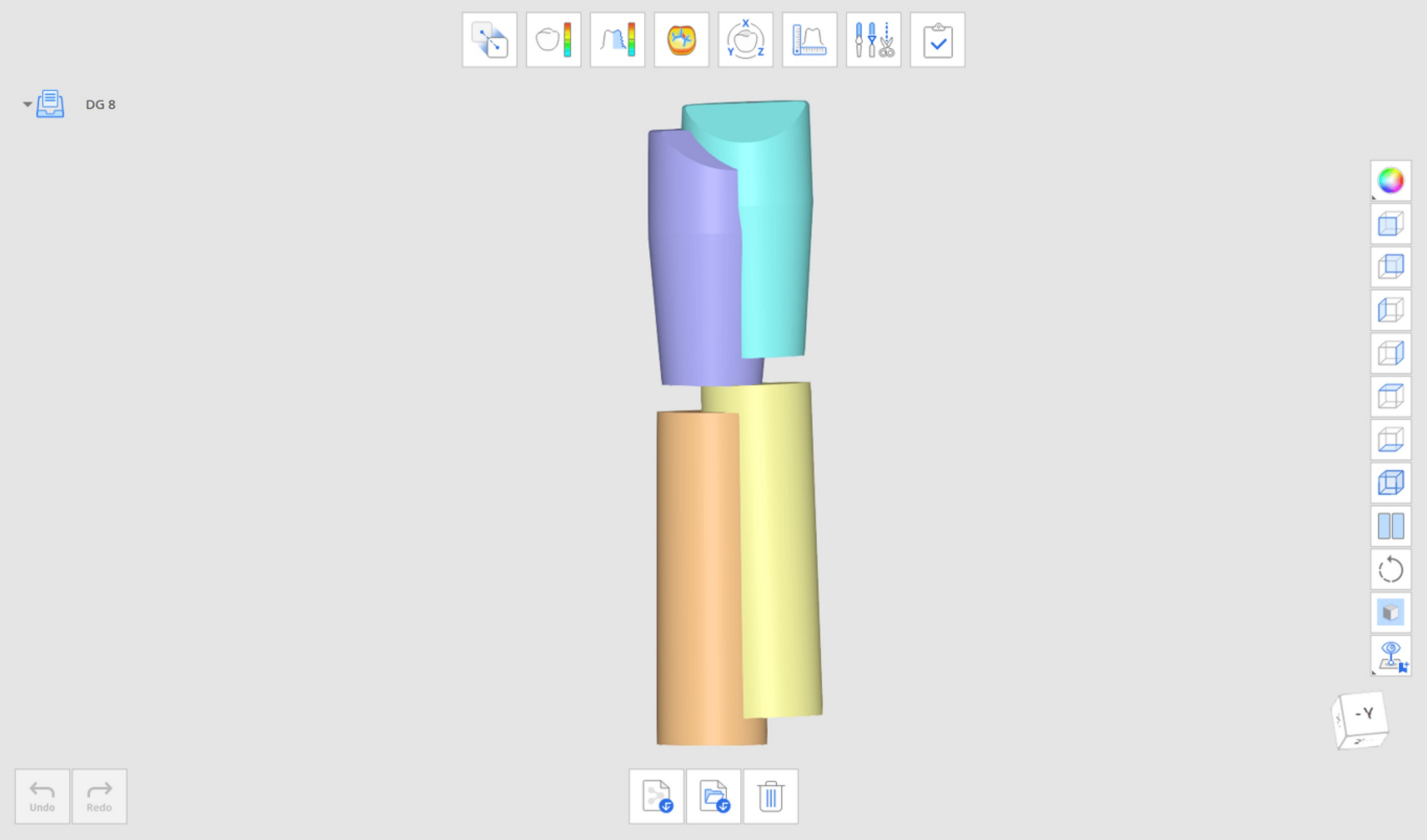
Reference cylinder (orange) and placed cylinder (yellow) with their corresponding scan bodies alignment on Medit Design

The implants were converted on BSP to cylinders having the same implant characteristics. Each STL file was individually exported to the Medit Link^®^ software (version 3.4.2, Medit, Seoul, South Korea), and using the Medit Design™ extension (version 2.1.4.97), the reference and placed implant scan bodies were aligned using the model teeth for best-fit matching. The two aligned scan bodies with their respective cylindrical shapes were isolated, one representing the placed implant and the other representing the planned implant (Figure 5).

**Figure 6:**
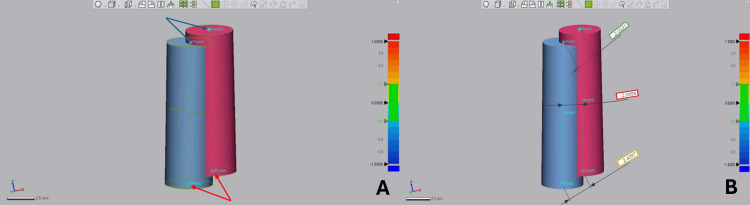
Reference cylinder (blue) and placed cylinder (pink) (A) Points 1 and 3 (blue arrows) at the center of cylinder platforms and points 2 and 4 (red arrows) at the center of cylinder apexes. (B) Three-dimensional coronal deviation (outlined in green), Δ angle (outlined in red), and 3D apical deviation (outlined in yellow) values given by the software.

To validate the 2D, 3D, and volumetric variable assessment methods, repeated measurements were performed on a subset of the sample to ensure measurement consistency and reproducibility using the intraclass correlation coefficient (ICC). Following reference cylinder and placed cylinder alignment, aligned cylinder STL files were imported into the Geomagic^®^ Control X software version 2022.1.0.7 (3D Systems, Rock Hill, SC) for 3D deviation assessment. Two points (points 1 and 3) at the middle of cylinder platforms and two points (points 2 and 4) at the center of cylinder apexes were defined, obtaining x, y, and z coordinates for each point (Figure 6A). Three-dimensional coronal deviation, defined as the discrepancy at the implant platform center in the x, y, and z plane, and 3D apical deviation, defined as the discrepancy at the implant apical part in the x, y, and z plane, values were obtained through the software. Angular deviation (Δ angle), referring to any divergence between central implant axes, was assessed after defining a vector for each implant joining its two points using the "Vector" module; Δ angle values were obtained by the software in degrees (°) (Figure 6B).

Two-dimensional deviation assessment included calculating the coronal linear deviation (CLD), coronal vertical deviation (CVD), apical vertical deviation (AVD), and apical linear deviation (ALD). CLD and ALD were defined, respectively, as the cylinder platform and apex deviation in the x and y. CVD and AVD were defined as the apico-coronal variation measured at the implant platform (CVD) and apex (AVD), respectively, following only the z axis. These variables, all expressed in mm, were assessed using the Euclidean distance formulas: CLD = $\sqrt{(x_{3}-x_{1})^{2} + (y_{3}-y_{1})^{2}}$, ALD = $\sqrt{(x_{4}-x_{2})^{2} + (y_{4}-y_{2})^{2}}$, CVD = $\sqrt{(z_{3}-z_{1})^{2}}$, and AVD = $\sqrt{(z_{4}-z_{2})^{2}}$.

**Figure 7:**
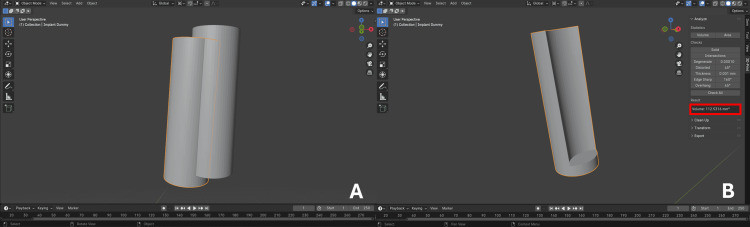
(A) Aligned implants displayed in the Blender software with the reference implant outlined in orange; and (B) the residual shape volume obtained after the subtraction of the common volume occupied by the two cylinders is outlined in red

To quantify the accuracy of each guided technique, the common volume occupied by the two cylinders was subtracted using "Boolean difference" in the Blender™ software version 4.0 (Blender Foundation, Amsterdam, Netherlands), and V_Residual shape_ in mm^3^ was obtained, representing the volume of the residual shape unoccupied by neither of the cylinders (Figures 7A, 7B). By using V_Residual shape_ and V_Cylinder_, each guidance technique's accuracy was quantified using the following formula: accuracy percentage = $100 -([V_{Residual\ shape} / V_{Cylinder}] \times 100)$.

The statistical analysis was conducted using RStudio version 2024.12.1 (Posit Software, Boston, MA). This study aimed to compare the accuracy of implant placement between static and dynamic guidance by analyzing eight deviation-related variables. Normality was assessed using the Shapiro-Wilk test. For variables that followed a normal distribution, the Welch t-test was used to compare static with dynamic guidance. For variables that did not meet the normal distribution, the Wilcoxon rank-sum test was applied instead. The 95% confidence interval (CI) for the mean difference was also calculated to provide an estimate of the precision of the observed differences. To assess intra-observer reliability, 30% of the samples were randomly re-evaluated by the same examiner using the same digital alignment and measurement workflow. The significance level was set at p ≤ 0.05.

## Results

Three-dimensional deviations

**Figure 8:**
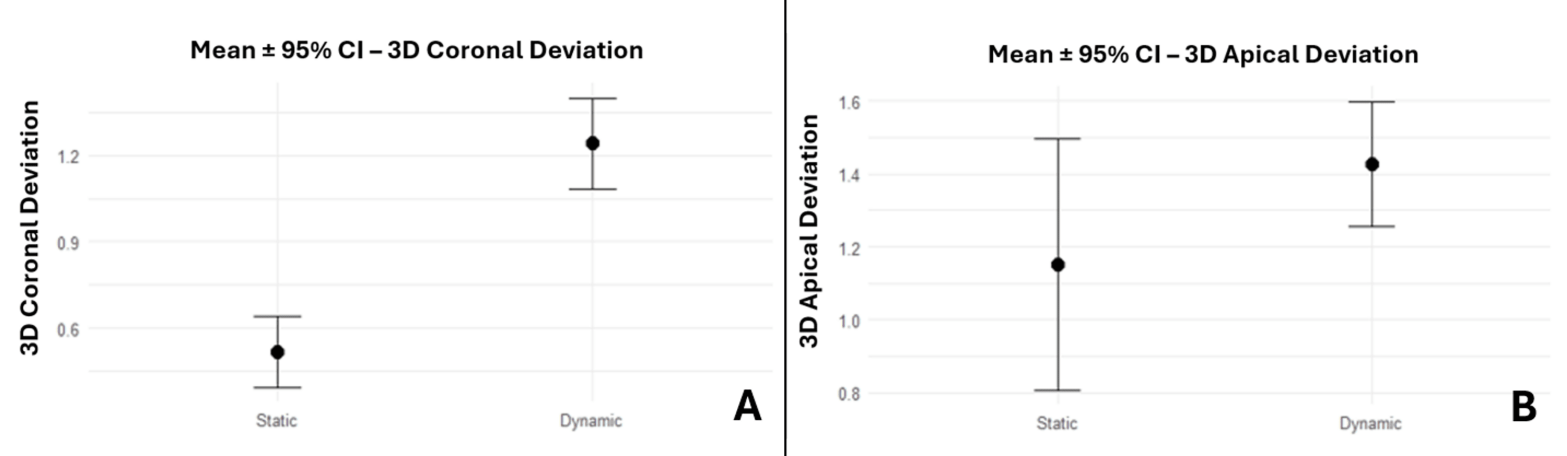
Bar plots displaying the comparison of s-GIP with d-GIP for 3D deviations, expressed as mean ± 95% CI (A) Bar plots displaying the comparison of s-GIP with d-GIP for 3D coronal deviation, expressed as mean ± 95% CI. (B) Bar plots displaying the comparison of s-GIP with d-GIP for 3D apical deviation, expressed as mean ± 95% CI. s-GIP: static-guided implant placement; d-GIP: dynamic-guided implant placement; CI: confidence interval.

The Welch t-test revealed that d-GIP demonstrated higher 3D coronal deviation compared to s-GIP with statistical significance (95% CI: 0.54, 0.91; p < 0.001). However, it did not reveal any statistical significance in terms of 3D apical deviation (95% CI: -0.09, 0.64; p = 0.131) between the two guidance techniques despite having a higher 3D apical deviation in d-GIP compared to s-GIP (1.43 mm and 1.15 mm, respectively) (Figure 8).

Two-dimensional deviations

**Figure 9:**
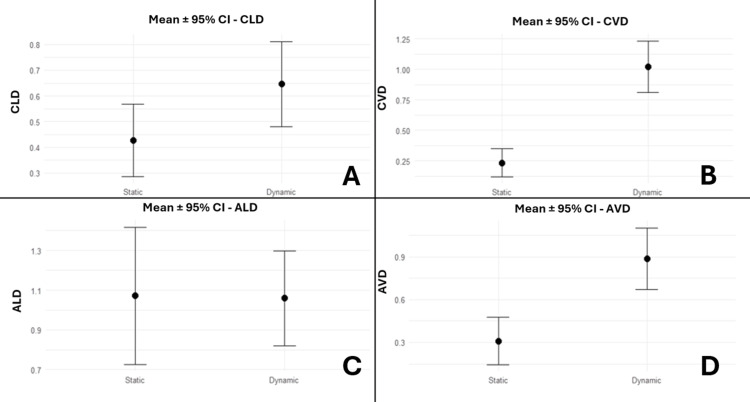
Bar plots displaying the comparison of s-GIP with d-GIP for 2D deviations, expressed as mean ± 95% CI (A) Bar plots displaying the comparison of s-GIP with d-GIP for coronal linear deviation (CLD), expressed as mean ± 95% CI. (B) Bar plots displaying the comparison of s-GIP with d-GIP for coronal vertical deviation (CVD), expressed as mean ± 95% CI. (C) Bar plots displaying the comparison of s-GIP with d-GIP for apical linear deviation (ALD), expressed as mean ± 95% CI. (D) Bar plots displaying the comparison of s-GIP with d-GIP for apical vertical deviation (AVD), expressed as mean ± 95% CI. s-GIP: static-guided implant placement; d-GIP: dynamic-guided implant placement; CI: confidence interval.

The Welch t-test revealed a statistically significant CLD and CVD (95% CI: 0.02, 0.42 (p = 0.036); 95% CI: 0.56, 1.02 (p < 0.001), respectively). However, ALD was not seen to be statistically significant (95% CI: -0.41, 0.38; p = 0.945). Since AVD did not follow normality, the Wilcoxon test was employed and revealed a statistically significant increase in d-GIP (95% CI: 0.3, 0.9; p = 0.001) (Figure 9).

Angular deviation and accuracy percentage

**Figure 10:**
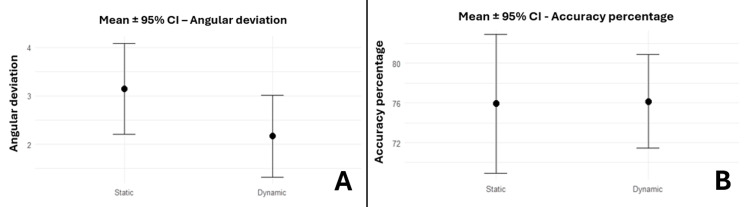
Bar plots displaying the comparison of s-GIP with d-GIP for angular deviation and accuracy percentage, expressed as mean ± 95% CI (A) Bar plots displaying the comparison of s-GIP with d-GIP for angular deviation, expressed as mean ± 95% CI. (B) Bar plots displaying the comparison of s-GIP with d-GIP for accuracy percentage, expressed as mean ± 95% CI. s-GIP: static-guided implant placement; d-GIP: dynamic-guided implant placement; CI: confidence interval.

Angular deviation and accuracy percentage (95% CI: -2.15, 0.2 (p = 0.097); 95 % CI: -7.7, 8.14 (p = 0.954), respectively) did not reach statistical significance, although angular deviation tended to be lower in the dynamic group (Figure 10).

Descriptive statistics (mean ± SD, median (IQR), and 95% CI) were computed for each parameter (Table 1).

**Table 1:** Descriptive statistics (mean ± SD, median (IQR), and 95% CI) by group *Significant. CLD: coronal linear deviation; CVD: coronal vertical deviation; ALD: apical linear deviation; AVD: apical vertical deviation; CI: confidence interval.

Variables	Group	Mean ± SD	Median (IQR)	95% CI	P-value
3D coronal deviation	Static	0.52 ± 0.17	0.51 (0.11)	0.54, 0.9	<0.001*
	Dynamic	1.24 ± 0.22	1.28 (0.34)		
3D apical deviation	Static	1.15 ± 0.48	1.23 (0.4)	-0.09, 0.64	0.131
	Dynamic	1.43 ± 0.24	1.42 (0.35)		
CLD	Static	0.43 ± 0.2	0.43 (0.33)	0.02, 0.42	0.036*
	Dynamic	0.65 ± 0.23	0.65 (0.23)		
CVD	Static	0.23 ± 0.16	0.24 (0.27)	0.56, 1.02	<0.001*
	Dynamic	1.02 ± 0.3	1.08 (0.51)		
ALD	Static	1.07 ± 0.48	1.1 (0.41)	-0.41, 0.38	0.945
	Dynamic	1.06 ± 0.34	1.08 (0.23)		
AVD	Static	0.31 ± 0.23	0.27 (0.29)	0.3, 0.9	0.001*
	Dynamic	0.89 ± 0.3	1.06 (0.45)		
Angular deviation	Static	3.15 ± 1.32	3.46 (1.38)	-2.15, 0.2	0.097
	Dynamic	2.17 ± 1.18	2.15 (1.45)		
Accuracy percentage	Static	75.9 ± 9.79	74.8 (8.45)	-7.7, 8.14	0.954
	Dynamic	76.1 ± 6.59	77 (10.1)		

Intra-observer reliability demonstrated good to excellent agreement across all variables. The overall ICC was 0.997 (95% CI: 0.995, 0.998). ICC values were 0.949 (95% CI: 0.899, 0.975) for 2D deviations, 0.886 (95% CI: 0.654, 0.966) for 3D deviations, and 0.912 (95% CI: 0.505, 0.987) for volumetric measurements. Overall, since statistically significant differences between s-GIP and d-GIP have been denoted, the null hypothesis (H_0_) was partially rejected.

## Discussion

Mispositioned implants are recognized as a primary etiological factor contributing to unfavorable biomechanical stress distribution, leading to biological and mechanical complications [17]. To improve surgical accuracy, GIP techniques were introduced, allowing implant placement according to virtual preoperative planning [12]. Although existing literature reports the superior positioning accuracy of GIP compared to FHIP [18], this advantage is often offset by higher treatment costs related to template fabrication and extended implant planning time [19]. Our study evaluated 2D, 3D, and volumetric deviations, and our findings allowed us to partially refute the null hypothesis (H_0_), indicating statistically significant differences in positioning deviations between s-GIP and d-GIP for 3D coronal deviation, CLD, CVD, and AVD. Three-dimensional apical deviation, ALD, angular deviation, and accuracy percentage were comparable.

The accuracy of GIP varies depending on the adopted techniques [20]. In the present study, both s-GIP and d-GIP yielded high levels of implant positioning accuracy. The static approach showed CVD and AVD mean values of 0.23 mm and 0.31 mm, respectively. In contrast, the d-GIP approach resulted in CVD and AVD values of 1.02 mm and 0.89 mm, respectively. These variables were statistically significant with p < 0.001 and p = 0.001 for CVD and AVD, respectively. Our findings corroborate those of Kang et al., who demonstrated that the d-GIP approach showed inferior accuracy when compared with s-GIP [20].

Among the evaluated parameters associated with s-GIP and d-GIP in an in vitro study by Mediavilla Guzmán et al., only the angular deviation exhibited a statistically significant difference between the two methods (p = 0.0272) [21]. However, despite having a lower mean angular deviation in d-GIP (2.17°) compared with s-GIP (3.15°), the mean difference in angular deviation between the two techniques was not statistically significant (p = 0.097). The difference in values observed in our study aligns with Vinnakota et al.'s systematic review and meta-analysis, in which d-GIP demonstrated less angular deviation compared with s-GIP, with this difference not being seen in clinical scenarios [22]. Furthermore, Stünkel et al. adopted a static pilot drilling protocol and compared it to the dynamic navigation system [23]. Their study demonstrated that mean tip, base, and angular deviation values were lower in s-GIP. Despite following a different methodology (fully guided s-GIP and pilot drill s-GIP) and a different variable assessment method (surface scan and CBCT), our results followed the same trend.

In terms of 2D and 3D deviations, 3D coronal (0.52 mm), 3D apical (1.15 mm), CLD (0.43 mm), and ALD (1.07 mm) values in static guidance were lower than in dynamic guidance, with 1.24 mm, 1.43 mm, 0.65 mm, and 1.06 mm, respectively. In our study, 3D coronal deviation and CLD reached the level of significance with p < 0.001 and p = 0.0036, respectively. However, 3D apical deviation and ALD were not seen to be statistically significant with p = 0.131 and p = 0.945, respectively. The discrepancies between our findings and those of Yimarj et al. might be due to the different settings in which the study took place (in vitroand in vivo), as factors affecting placement accuracy are easier to handle inin vitrosettings, as well as considering the small sample size of 20 implants [24].

To our knowledge, our study is the first to quantify accuracy using implant volume. The volumetric discrepancy between planned and placed implants allowed us to quantify the accuracy for each technique. Our findings demonstrated that the accuracy percentage was slightly higher in the d-GIP (76.1%) compared with the s-GIP (75.9%), although no statistical difference in the volumetric deviation between s-GIP and d-GIP (p = 0.954) was noted.

In light of these findings, the null hypothesis (H_0_) is partially rejected within this in vitro study's limitations. Although guided surgery offers significant potential to improve the accuracy of implant placement, inconsistencies in achieving the perfect implant planned position remain a persistent challenge, as shown in our study. In s-GIP, these discrepancies are likely explained by multiple factors, including deviations introduced during the drilling process and limitations related to guide stability, seating, or fit during its use [25]. However, in d-GIP, these deviations may be attributed to potential calibration-related issues [26], and focusing on the dynamic navigation system monitor display during implant site preparation remains challenging [20].

While static-guided systems are generally accurate, they may be more prone to issues such as the inability to make intraoperative adjustments, guide misfit, drill-sleeve tolerance, inaccuracies in 3D-printed surgical guides, or slight discrepancies in seating. These issues are particularly relevant in the posterior region of the mouth and in patients with limited mouth opening, often requiring the clinician to angulate the drill [16]. This aligns with findings of the ITI consensus publication, indicating that errors at each phase of the digital workflow, ranging from data acquisition and planning to implant placement execution, can independently or cumulatively contribute to inaccuracies. The cumulative effect of small deviations at each stage can lead to an overall inaccuracy in implant positioning [27].

This study has limitations, primarily being an in vitro study with a small sample size (n = 20), which possibly contributed to the lack of significance in some comparisons. Although appropriate for exploratory analysis, the limited sample size may have reduced statistical power and increased the risk of type II error. Therefore, nonsignificant findings should be interpreted with caution, and further studies with larger samples are warranted. Given these constraints, a larger sample with models simulating actual bone anatomy and gingival simulation would allow a more detailed assessment of various parameters, leading to more reliable analyses. A common limitation to all studies evaluating software, hardware, and technology-based dental procedures is the continuous advancement and evolution of digital technology, which could positively or negatively influence the accuracy of static and dynamic systems adopted in our study. For instance, it is noteworthy to consider these evolving technologies when extrapolating our findings, especially when comparing our utilized procedures to novel techniques that employ different materials, printer technologies, and dynamic systems.

## Conclusions

The study findings indicated that both s-GIP and d-GIP showed high levels of accuracy when assessing overall implant deviations, suggesting a potential advantage of these techniques over FHIP in achieving more precise implant positioning. However, given the controlled nature of this in vitro study, further validation of these study outcomes is required before extrapolating to clinical practice. These findings have important clinical implications, as they provide preliminary information on technique accuracy under in vitroconditions; nonetheless, additional clinical studies are needed to confirm these results and evaluate their applicability in real-world clinical scenarios.
